# Involvement Modulates the Effects of Deception on Memory in Daily Life

**DOI:** 10.3389/fpsyg.2021.756297

**Published:** 2021-10-15

**Authors:** Yan Li, Zhiwei Liu

**Affiliations:** ^1^Faculty of Psychology, Tianjin Normal University, Tianjin, China; ^2^School of Education and Psychology, Sichuan University of Science & Engineering, Zigong, China

**Keywords:** involvement, false denial, denial-induced forgetting, memory, daily life

## Abstract

Previous studies have demonstrated that liars who adopt a false denial strategy often forget what they lied about, which has been labeled the denial-induced forgetting (DIF) effect. However, several investigations have not found such an effect. It has been suggested that involvement might play a role in the inconsistency. The present study was designed to directly determine whether involvement modulates the effects of deception on memory. Participants were assigned randomly to either high- or low-involvement conditions and were required to complete a mock shopping task. They were then asked to participate in an interview in which they were asked to respond honestly or deceptively. Two days later, final memory tests were given, and the participants were asked to give honest responses. We found a DIF effect in the high-involvement condition but not in the low-involvement condition. Moreover, the liars in the high-involvement condition created more non-believed memories in the source memory test and the destination memory test than the honest participants. In addition, liars in both the high- and low-involvement conditions forgot who they lied to. We conclude that the effects of deception on memory could be influenced by the degree of involvement.

## Introduction

Deception, or lying, is a kind of behavior that aims to provide false information and induce a false belief (Hyman, 1989). Lying can have negative consequences, especially for the liar's memory. A wealth of studies have consistently demonstrated that liars' memories can be disrupted by their lies (Otgaar et al., 2014a, 2018a, 2020; Romeo et al., 2019b; Battista et al., 2020a, 2021a). People might forget some details of events that they have lied about (Otgaar et al., 2018a; Romeo et al., 2019b; Battista et al., 2020a), and they may also forget what they lied about during an interview (Battista et al., 2020a,b; Battista et al., 2021a). In short, one's memory can be impaired after one lies.

False denial is a type of deception, and it also affects the deceiver's memory. The following procedure was used to study the effects of false denial on memory: participants were presented with a video or were asked to perform a task. They were then randomly assigned to either an honest condition or a false denial condition and engaged in an interview. Questions about the video or event are asked in the interview. The participants in the honest condition are directed to respond honestly, and those in the false denial condition are asked to give negative responses to all questions. Then, a source memory test, in which the participants are asked to identify the items whether they were asked about in the interview, is administered several days later, and the participants are asked to respond honestly. Comparing the performance between the groups in the source memory test, many studies in this field have found that participants in the false denial group lose more memories of what has been asked about in the interview than those in the honest group (Otgaar et al., 2014a, 2016, 2018a, 2020; Romeo et al., 2019a; Battista et al., 2021a). The pattern of results found in the source memory test has been considered to constitute a denial-induced forgetting (DIF) effect, which explains why participants in the false denial condition forget more interview details.

Some studies, however, have failed to find a DIF effect. Romeo et al. (2019b) asked participants to complete a mock crime task in which the participants were asked to sneak into a professor's lab, get the password to the professor's email account, and then steal the answers to a statistics exam from his email. The participants were randomly assigned to an honest condition, a false denial, or a simulated amnesia condition. An interview was administered after the mock crime. In the interview, the participants in the honest condition were asked to respond honestly, those in the false denial condition were asked to give negative responses, and those in the simulated amnesia condition were asked to simulate memory loss (e.g., respond “I do not remember” or “My memory is vague”). A day later, the participants were given a source memory test, in which they were directed to respond honestly. Data analysis revealed no significant effects between the false denial and honest groups in the source memory test, suggesting that no DIF effect was found in the study. Combining this finding with those from previous studies that obtained a DIF effect, the authors suggested that there may be a boundary to the DIF effect, and the effect would disappear when the participants are actively involved in the event. This suggestion might be correct because another study also did not find a DIF effect. In their study, Li and her colleagues (Li et al., 2021b) asked the participants to engage in a mock shopping task, and the participants were required to choose to lie or be honest in an interview. Forty-eight h after the interview, the participants took a source memory test, in which they were asked to identify whether the presented items were asked about in the interview. No significant effect was found for the items on the shopping list and asked about in the interview between the groups, suggesting that no DIF effect occurred. However, another study from Li and her colleagues (Li et al., 2021a), using the same paradigm as their previous study (Li et al., 2021b) but randomly assigning the participants to honest or deception conditions, found a DIF effect. Therefore, whether the DIF effect has a boundary and whether the appearance of the DIF effect is related to the degree of involvement remain unclear.

The present study was carried out with the aim of directly verifying whether involvement modulates the DIF effect. It is hoped that this study will contribute to a deeper understanding of the effects of deception on memory. It has been suggested that the effects of deception on memory depend on how many cognitive resources are needed during lying (Otgaar and Baker, 2018). That is the more cognitive resources that are required by the deceptive strategy used by a liar, the more memory disruption the liar will experience. However, whether other variables, such as the degrees of involvement, might modulate the effects of deception on memory is still unknown. Moreover, previous studies in this field have often presented a video or images to the participants or asked the participants to complete a mock task. However, people often tell lies about events they have heard about from someone else in daily life. In this case, liars do not see anything or feel anything about the event, and they have no involvement in the events they lie about. Whether liars' memories are disrupted when they are not involved in the event they lie about remains unknown. These issues will be investigated in this study. Finally, the present study also paid attention to destination memory, which has been ignored in most previous studies. Destination memory is the memory for the people who were previously provided information (Marsh and Hicks, 2002). For liars, destination memory is memory about the person to which they lied. Liars need to remember the people they lie to or risk inconsistency that may reveal their lies. Whether liars' destination memories are affected by their lies was also studied in the present study.

A multiple factorial design was used in the present study. The participants were randomly assigned to either high- or low-involvement conditions and were then asked to engage in a mock shopping task. The participants were also required to respond deceptively or be honest in an interview. Two days later, the participants were needed to complete the final memory tests and respond honestly. Comparisons in the memory tests between groups were conducted to determine whether the effects of deception on memory would be modulated by involvement. We expected that the effects of deception on memory would differ based on the degree of involvement.

## Methods

The present study was approved by the research ethics committee of the Faculty of Psychology at Tianjin Normal University and conducted following the Declaration of Helsinki principles.

### Participants

Using G^*^Power (version 3.1.9.7) with a medium effect size (*f* = 0.3) and a power of 0.8, a total sample size of 90 participants was required. One hundred college students (21 male and 79 female) aged 18–25 (*M* = 20.62 years, *SD* = 1.48) were recruited. Five participants failed to join session 2 due to personal reasons. Therefore, 95 participants completed this study (low-involvement: 23 participants in both the honest and deception groups; high-involvement: 24 and 25 participants in the deception and honest group, respectively). All participation was voluntary, and all participants gave their written informed consent following the Helsinki Declaration. The participants were paid 35 yuan for their participation.

### Design and Procedure

A between-subject 2 (response: deception, honest) ×2 (involvement: high, low) factorial design was used in this study. Therefore, the experiment included four conditions, and the participants were assigned to each condition randomly.

A small store was set up on the third floor of our institute building. Twenty kinds of goods were provided for sale: bottled water, cola, bread, chocolate, seaweed, strawberry pie, chewing gum, instant coffee, instant noodles, cookies, garbage bags, tissue, towel, hangers, soap, N95 mask, toothpaste, toothbrush, cotton swab, and laundry detergent.

This study used a daily-life paradigm and was divided into two sessions. In session 1, the participants were asked to complete a mock shopping task for three min. The participants in the high-involvement condition were required to go shopping in the small store and buy 10 products, and they did not need to pay for them. The participants in the low-involvement condition were directed to imagine they were shopping in a store and to mark 10 goods they wanted to buy on a written list that contained the 20 kinds of goods sold in the small store. After shopping, all of the participants were asked to complete a 5-min filler task (playing Tetris).

Following the filler task, a baseline memory test was given. In this test, the participants were asked to free recall what items they bought a few min earlier. Moreover, they were also required to indicate their memory (Do you actually remember that you bought this item: 1 = *no memory at all*, 8 = *clear and complete memory*) and belief (How strong is your belief that you bought this item: 1 = *no belief* , 8 = *strong belief* ) for each item. These scales were derived from the Autobiographical Belief and Memory Questionnaire (Scoboria et al., 2004, 2014), which has been validated and widely used in this field (Otgaar et al., 2016, 2018a). After finishing the baseline memory test, the participants engaged in another filler task (playing Tetris) for 5 min.

Then, the participants were told two people on the second floor wanted to know what they bought and would ask them some questions in an interview. The interviewers were female and were strangers to the participants, and they received the shopping list while the participants were completing the baseline memory test. Ten items were prepared for the interview: five items were randomly selected from the shopping list, and five items were not sold in the store. A fixed question structure (Did you buy XXX?) was used in the interview. The two interviewers asked questions alternately. To make the destination memory easier for the participants, an interviewer asked about the items on the shopping list, and another asked about the items not sold in the store. The participants in the honest group were required to answer all the questions honestly, and those in the deception group were needed to give deceptive responses to all the questions. All the participants were asked to respond as accurately as possible based on the instructions.

The participants took part in session 2 48 h after the interview. Three memory tests were administered in a fixed order in this session. First, the participants were needed to finish an item memory test, in which they were asked to free recall the items they bought two days ago. Moreover, the participants were also required to indicate their memory (Do you actually remember that you bought this item: 1 = *no memory at all*, 8 = *clear and complete memory*) and belief (How strong is your belief that you bought this item: 1 = *no belief* , 8 = *strong belief* ) for each item. Then, a source memory test was administered. Twenty items were randomly presented in the source memory test: five items that were on the shopping list and were asked about in the interview, five items that were not sold in the store but were asked about in the interview, five items that were on the shopping list but were not asked about in the interview, and five items that were not sold in the store and were not asked about in the interview. The participants were told to report whether the items were asked about in the interview. Moreover, they were asked to indicate their memory (Do you actually remember that this item was/was not asked in the interview: 1 = *no memory at all*, 8 = *clear and complete memory*) and belief (How strong is your belief that this item was/was not asked about in the interview: 1 = *no belief* , 8 = *strong belief* ) for each item. Finally, the participants took a destination memory test, in which two photos of the interviewers and the 10 items that were asked about in the interview were presented. The participants needed to determine which interviewer asked the question for each item. The participants were also required to indicate their memory (Do you actually remember that this is the person who asked you about this item: 1 = *no memory at all*, 8 = *clear and complete memory*) and belief (How strong is your belief that this is the person who asked you about this item: 1 = *no belief* , 8 = *strong belief* ) for each item.

## Results

It has been argued that the analysis of variance (ANOVA) *F*-test is not informative of the source of an interaction or main effect when the factorial experiments have more than two levels, but the linear (mixed) models are (Schad et al., 2020). Therefore, we used the lme4 package (Bates et al., 2015) to analyze all the data in the R system (R Development Core Team, 2016). With participants and items as crossed random effects, a linear mixed-effects model (LMM) and a generalized linear mixed-effects model (GLMM) (Baayen et al., 2008) were used to analyze belief and memory ratings and response accuracy, respectively. Following convention, *t* or *z* values larger than 1.96 were considered statistically significant for two-tailed.

Descriptive and inferential statistics are shown in Tables 1, 2.

**Table 1:** Mean error rates and ratings for correct responses in the memory tests for each condition.

		**High-involvement**		**Low-involvement**	
		**Honest**	**Deception**	**Honest**	**Deception**
Baseline memory test	Error Rates (%)	10.80 (1.98)	11.67 (2.08)	11.74 (2.13)	10.01 (1.98)
	Memory Ratings	6.97 (0.1)	7.37 (0.07)	7.38 (0.08)	7.43 (0.08)
	Belief Ratings	7.74 (0.05)	7.88 (0.04)	7.74 (0.05)	7.73 (0.06)
Item memory test	Error Rates (%)	11.6 (2.03)	10.83 (2.01)	12.17 (2.16)	10.87 (2.06)
	Memory Ratings	6.35 (0.12)	6.72 (0.12)	7.09 (0.11)	7.08 (0.12)
	Belief Ratings	7.61 (0.07)	7.6 (0.09)	7.39 (0.1)	7.41 (0.09)
Source memory test	Error Rates (%)	25.4 (1.95)	35.83 (2.19)	28.48 (2.11)	31.96 (2.18)
	Memory Ratings	5.45 (0.12)	6.24 (0.11)	6.38 (0.1)	5.81 (0.12)
	Belief Ratings	6.02 (0.12)	6.03 (0.12)	6.29 (0.12)	5.84 (0.13)
Item category 1	Error Rates (%)	15.2 (3.22)	30.83 (4.23)	30.44 (4.31)	29.57 (4.27)
	Memory Ratings	5.98 (0.2)	6.94 (0.14)	6.49 (0.22)	6.09 (0.2)
	Belief Ratings	6.53 (0.2)	6.77 (0.16)	6.56 (0.23)	6.1 (0.2)
Item category 2	Error Rates (%)	32 (0.04)	32.5 (0.04)	33.33 (0.04)	35.65 (0.04)
	Memory Ratings	5.99 (0.22)	6.25 (0.2)	6.61 (0.17)	6.39 (0.2)
	Belief Ratings	6.53 (0.21)	6.02 (0.23)	6.32 (0.22)	6.5 (0.22)
Item category 3	Error Rates (%)	43.2 (0.04)	52.5 (0.05)	39.13 (0.05)	46.09 (0.05)
	Memory Ratings	4.34 (0.29)	5.44 (0.27)	5.41 (0.23)	4.6 (0.3)
	Belief Ratings	5.06 (0.29)	5.35 (0.28)	5.26 (0.27)	4.4 (0.31)
Item category 4	Error Rates (%)	11.2 (2.83)	27.5 (4.09)	11.3 (2.97)	16.52 (3.48)
	Memory Ratings	5.25 (0.24)	6.1 (0.22)	6.71 (0.17)	5.92 (0.22)
	Belief Ratings	5.76 (0.24)	5.78 (0.25)	6.7 (0.19)	5.99 (0.22)
Destination memory test	Error Rates (%)	23.2 (2.68)	45.42 (3.22)	30 (3.03)	45.22 (3.29)
	Memory Ratings	4.93 (0.17)	5.34 (0.19)	5.34 (0.19)	4.01 (0.2)
	Belief Ratings	5.88 (0.17)	5.1 (0.22)	5.45 (0.2)	3.97 (0.21)

*The standard error of the mean is shown in parentheses. Item Categories 1, 2, 3 and 4 are items in the source memory test. Item Category 1, items that were on the shopping list and were asked about in the interview; Item Category 2, items that were not sold in the store and were asked about in the interview; Item Category 3, items that were on the shopping list and were not asked about in the interview; and Item Category 4, items that were not sold in the store and were not asked about in the interview*.

**Table 2:** Statistical effect for memory tests.

		**Error rates**			**Memory Ratings**			**Belief Ratings**		
		***b***	***SE***	***z***	***b***	***SE***	***t***	***b***	***SE***	***t***
Baseline memory test	Intercept	2.22	0.21	10.58	7.27	0.08	88.06	7.77	0.04	220.7
	Involvement (I)	0.01	0.21	0.03	0.21	0.15	1.37	0.07	0.07	1.01
	Response (R)	0.02	0.21	0.1	0.22	0.15	1.49	0.07	0.07	1
	I × R	0.28	0.42	0.66	0.34	0.3	1.12	0.16	0.14	1.13
Item memory test	Intercept	2.16	0.21	10.12	6.81	0.13	51.62	7.5	0.07	113.13
	Involvement (I)	0.13	0.21	0.64	0.55	0.24	**2.23**	0.21	0.12	1.73
	Response (R)	0.19	0.21	0.91	0.17	0.24	0.69	0.01	0.12	0.04
	I × R	0.21	0.42	0.51	0.29	0.49	0.6	0.02	0.24	0.07
Source memory test	Intercept	0.82	0.1	8.43	5.92	0.14	43.76	6	0.13	44.9
	Involvement (I)	0	0.1	0.04	0.2	0.25	0.81	0.03	0.24	0.13
	Response (R)	0.33	0.1	**3.24**	0.17	0.25	0.68	0.15	0.24	0.63
	I × R	0.38	0.2	**1.86**	1.29	0.49	**2.6**	0.46	0.49	0.94
Item category 1	Intercept	1.18	0.15	7.89	6.33	0.17	36.65	6.47	0.16	39.22
	Involvement (I)	0.45	0.28	1.63	0.2	0.29	0.7	0.32	0.28	1.14
	Response (R)	0.47	0.28	1.7	0.39	0.29	1.36	0.01	0.28	0.04
	I × R	1.03	0.55	**1.86**	1.22	0.57	**2.14**	0.64	0.57	1.12
Item category 2	Intercept	0.71	0.11	6.63	6.3	0.22	28.52	6.34	0.21	30.58
	Involvement (I)	0.1	0.21	0.5	0.38	0.26	1.45	0.17	0.27	0.63
	Response (R)	0.06	0.21	0.31	0.08	0.26	0.3	0.12	0.27	0.46
	I × R	0.08	0.42	0.2	0.45	0.53	0.85	0.65	0.54	1.21
Item category 3	Intercept	0.3	0.17	1.8	4.99	0.2	24.63	5.03	0.22	22.77
	Involvement (I)	0.24	0.26	0.95	0.19	0.41	0.47	0.3	0.42	0.71
	Response (R)	0.4	0.26	1.55	0.1	0.41	0.26	0.19	0.42	0.45
	I × R	0.14	0.51	0.27	1.83	0.81	**2.25**	1.17	0.84	1.38
Item category 4	Intercept	2.03	0.23	8.68	5.98	0.18	33.32	6.03	0.18	33.61
	Involvement (I)	0.36	0.36	0.99	0.68	0.36	**1.9**	0.6	0.36	1.66
	Response (R)	0.91	0.37	**2.46**	0.09	0.36	0.25	0.27	0.36	0.74
	I × R	0.75	0.73	1.03	1.6	0.72	**2.23**	0.68	0.72	0.95
Destination memory test	Intercept	0.7	0.11	6.15	4.81	0.19	25.05	4.82	0.21	23.12
	Involvement (I)	0.2	0.22	0.9	0.4	0.36	1.11	0.72	0.41	**1.76**
	Response (R)	0.98	0.22	**4.32**	0.49	0.36	1.34	0.98	0.41	**2.4**
	I × R	0.4	0.45	0.89	1.56	0.73	**2.14**	0.43	0.82	0.53

*Bold numbers indicate significant values. Item Categories 1, 2, 3 and 4 are items in the source memory test. Item Category 1, items that were on the shopping list and were asked about in the interview; Item Category 2, items that were not sold in the store and were asked about in the interview; Item Category 3, items that were on the shopping list and were not asked about in the interview; and Item Category 4, items that were not sold in the store and were not asked about in the interview*.

### Baseline Memory Test

As shown in Table 2, no significant differences in main or interaction effects were found in the baseline memory test, suggesting that the differences in baseline memory test between the honest and deception groups, both in high- and low-involvement conditions, did not reach statistical significance in error rates and ratings.

### Item Memory Test

As shown in Table 2, only a significant difference in memory ratings was found, suggesting that the participants in the low-involvement condition had greater ratings than those in the high-involvement condition. No other significant differences were found in the item memory test. Those findings suggest no evidence of a difference in the effects of deception on item memory between high- and low-involvement conditions.

### Source Memory Test

A significant interaction effect in error rates and memory ratings was found. Analyses of simple effect showed that the participants in the low-involvement condition had no significant differences in error rates (*b* = 0.14, *SE* = 0.15, *z* = 0.32) or memory ratings (*b* = 0.48, *SE* = 0.32, *t* = 1.49) across groups. In the high-involvement condition, however, the participants in the honest group had lower error rates (*b* = 0.52, *SE* = 0.14, *z* = 3.62) and memory ratings (*b* = 0.82, *SE* = 0.37, *t* = 2.2) than those in the deception group. These findings suggest that the effects of deception on source memory were only observed in the high-involvement condition and were different between high- and low-involvement conditions.

Separate analyses were conducted to determine whether the performance differed across groups based on the item categories. As shown in Table 2, for the items that were on the shopping list and asked about in the interview, significant interaction effects in error rates and memory ratings were found. Simple effects analysis indicated no differences in error rates (*b* = 0.04, *SE* = 0.37, *z* = 0.1) or memory ratings (*b* = 0.24, *SE* = 0.45, *t* = 0.55) across groups were found in the low-involvement condition. In the high-involvement condition, the participants in the deception group had more significant error rates (*b* = 1, *SE* = 0.41, *z* = 2.42) and memory ratings (*b* = 0.97, *SE* = 0.38, *t* = 2.57) than those in the honest group. For the items that were not on the shopping list but were asked about in the interview, no significant effects in any dependent variables were found. For the items that were on the shopping list but were not asked about in the interview, a significant interaction effect in memory ratings was found. Simple analysis showed no effect between groups in the low-involvement condition (*b* = 0.78, *SE* = 0.56, *t* = 1.4), and the participants in the honest group had lower memory ratings than those in the deception group (*b* = 0.52, *SE* = 0.38, *t* = 2.1) under the high-involvement condition. For the items that were not on the shopping list and were not asked about in the interview, a main effect for response on error rate and an interaction effect on memory rating were found. The participants in the honest group made fewer errors than those in the deception group. Moreover, no effect was found between groups in memory ratings in the low-involvement condition (*b* = 0.72, *SE* = 0.44, *t* = 1.65), but the participants in the deception group had greater memory ratings than those in the honest group (*b* = 0.89, *SE* = 0.57, *t* = 2.12) under the high-involvement condition. Separate analyses revealed that the effects of deception on source memory observed in the high-involvement condition differed by the effects found in the low-involvement condition.

### Destination Memory Test

A main effect for response in error rates and belief ratings was found. The participants in the deception group made more errors than those in the honest group in identifying the targets they lied to, regardless of whether they had higher or lower involvement in shopping. The participants in the honest group had greater belief ratings than those in the deception group. Moreover, the main effect for involvement in belief ratings was found. The participants in the low-involvement condition had lower belief ratings than those in the high-involvement condition. In addition, we also found an interaction effect in memory ratings. Simple effects analyses indicated that participants in the honest group showed higher ratings than those in the deception group (*b* = 1.28, *SE* = 0.51, *t* = 2.53) under the low-involvement condition, and no effect was found between groups (*b* = 0.29, *SE* = 0.52, *t* = 0.56) in the high-involvement condition. These observations suggest that the effects of deception on destination memory in memory ratings but not in accuracy were different between the high- and low-involvement conditions.

### Non-believed Memories

Memories for which the belief ratings were at least two scale points lower than the memory ratings were classified as non-believed memories, regardless of whether the responses were correct (Clark et al., 2012; Otgaar et al., 2016). The number of non-believed memories in memory tests for each condition is shown in Table 3. Chi-square analyses were conducted to determine whether deception created more non-believed memories than honest. We found that the participants in the deception group created more non-believed memories than those in the honest group under the high-involvement condition, both in the source memory test (χ^2^ = 20.19, *p* <0.001) and the destination memory test (χ^2^ = 20.35, *p* <0.001). Other comparisons did not reach statistical significance. The findings observed in non-believed memories suggest that the effects of deception on memory differed in the high- and low-involvement conditions.

**Table 3:** The number of non-believed memories in the memory tests for each condition.

	**High-involvement**		**Low-involvement**	
	**Honest**	**Deception**	**Honest**	**Deception**
Baseline memory test	2	4	4	4
Item memory test	0	6	6	8
Source memory test	13	45	33	22
Destination memory test	5	30	29	17

## Discussion

People often deny the events or details of events that occur in daily life. Some studies have demonstrated that false denial could disrupt liar's memories and lead to the loss of memory about what they have lied about (Otgaar et al., 2018a, 2020; Battista et al., 2020a, 2021a), and this phenomenon is called the DIF effect (Otgaar et al., 2016). However, several studies have failed to obtain such an effect (Romeo et al., 2019b; Li et al., 2021b). Therefore, the present study was carried out to determine whether involvement is a variable that modulates the appearance of the DIF effect. In line with our expectations, the DIF effect depends on the degree of involvement. The DIF effect disappears when liars are less involved in the event but appears when liars are highly involved in the event. Therefore, there seems to be a boundary to the DIF effect, and it could be modulated by the degrees of involvement.

Two important issues need to be discussed in the present study. The first one is why false denial induces forgetting. This issue has been explained as false denial requiring cognitive resources so that “fewer resources were available to memorize the details that had been talked about during the interview” (Otgaar et al., 2018a). However, we slightly disagree with this explanation. In this study, the participants in the deception group under the high-involvement condition were asked to respond deceptively to all questions in the interview. Ten items were asked about during the interview: five items were on the shopping list, and five items were not sold in the store. Thus, the participants in the deception group needed to falsely deny (respond with “No, I did not buy XXX”) that they purchased the items on the shopping list and fabricate (“Yes, I bought XXX”) responses to items that were not available in the store. It has been suggested that fabrication requires substantially more cognitive resources than false denial (Otgaar and Baker, 2018). If the DIF effect results from having fewer cognitive resources available for remembering interview details, then fabrication would cause a greater forgetting effect. However, fabrication did not induce any forgetting effect (see the result of item category 2 in Tables 1, 2). Therefore, the DIF effect is probably independent of cognitive resource consumption.

We prefer another explanation that concerns the inhibition processes. Activation-decision-construction-action theory (ADCAT) has been proposed to explain the cognitive processes of deception (Walczyk et al., 2003, 2014). Memories of the truth, consisting of episodic memory and/or semantic memory, are encoded and stored in the long-term memory (LTM) system. The truth will be activated automatically by a clue or a question, and it will be transferred from LTM to working memory. Then, the potential liar will decide whether to respond honestly or deceptively. Once they decide to lie, the truth that is active in working memory will be inhibited by the decision to lie. Then, the liar constructs the lie in their mind and finally lies. Thus, it is important for liars to inhibit the truth before they tell lies. Successfully inhibiting the truth is a crucial step in lying. It has been suggested that inhibition is the underlying mechanism of memory disruption effects (Anderson and Green, 2001; Murayama et al., 2014). Liars who apply the false denial strategy need to inhibit the truth, which contains episodic and/or semantic information, and then give a negative response. Inhibiting and denying the truth might also inhibit the memory for the interview and result in liars failing to retrieve the memory of the interview and then induce a forgetting effect (Otgaar et al., 2018a). Unlike liars who use the false denial strategy, it is more important for those who use the fabrication strategy to construct an event or details that have not happened. Fabricators need to not only inhibit the truth but also construct permissible and plausible lies. It is more difficult and requires more cognitive resources to fabricate lies than false denial (Otgaar and Baker, 2018). Fabricated lies contain some new semantic and/or episodic information, which may help the liars remember that they have talked about these details in an interview. Thus, fabrication does not induce a forgetting effect.

The second issue that needs to be discussed is why involvement modulates the DIF effect. It has been suggested that liars need to inhibit the truth that is automatically activated from the LTM and is active in working memory (Walczyk et al., 2003, 2014). Moreover, inhibiting and denying the truth might inhibit memories of an interview (Otgaar et al., 2018a). In short, inhibition causes the DIF effect. In the present study, the participants in the high-involvement condition went shopping in a real store, and they could obtain semantic, visual, tactile, and episodic information while shopping. These pieces of information were interconnected to form the memory of the truth. On the other hand, the participants in the low-involvement condition “went shopping” on a piece of paper, and there was nothing but words on the paper. They obtain only some semantic information while shopping. Therefore, the memory of the truth contained much more complicated information for the participants in the high-involvement condition than for those in the low-involvement condition. On the other hand, the items used are very common in daily life, and the participants were very familiar with them. It would be very easy for the participants in the low-involvement condition to inhibit semantic information during the interview. Thus, more cognitive efforts might be required to inhibit the truth for the participants in the high-involvement condition than those in the low-involvement condition. The significant interaction effects observed in memory ratings in the source memory test and some item categories could be considered evidence for inhibition theory. In the high-involvement condition, the liars had higher memory ratings than the honest ones in source memory test. Therefore, the DIF effect was only observed in the high-involvement condition, possibly because more inhibition effort was needed for the liars during the interview.

However, previous studies in which participants were highly involved in a mock crime or shopping task have not obtained a DIF effect in the items or details that were denied (Romeo et al., 2019b; Li et al., 2021b). There are several possible explanations for this discrepancy. Romeo et al. (2019b) asked participants to complete a mock crime task in their study. The crime task was completely different from the shopping task, watching a crime video and viewing images. Crime is a behavior that violates the law. The questions asked in the interview pertained to the details of the criminal behavior and concerned some crime-relevant items. A previous study using a virtual mock crime task found that the criminal group had stronger peak amplitudes to the crime-relevant stimulus than the innocent group (Hahm et al., 2009), suggesting that the participants in the criminal group had greater neural responses to the crime-relevant items than those in the innocent group. Therefore, inhibiting and denying the truth did not impair their memory of the interview because the participants in the false denial group were more sensitive to the criminal details discussed in the interview. The underlying mechanism of false denial differs from simulated amnesia, and this may be why a DIF effect was found in the latter condition in their study (Romeo et al., 2019b). On the other hand, consistent with Li et al. (2021b), the present study also used a mock shopping task, but the former study failed to obtain a DIF effect. In their study (Li et al., 2021b), the participants were told that they could choose to respond deceptively or honestly during the interview, and the participants in the deception group chose to tell lies. The participants who chose to tell lies may have greater motivation and a higher tendency to deceive than those who chose to respond honestly, and these individual differences between the groups may have played a key role in failing to observe a DIF effect. However, in the present study, the participants were assigned randomly to deception or honest groups, and thus the individual differences were well-controlled.

In this study, we found that the participants in the deception groups, regardless of the high- or low-involvement conditions, lost more memories about the person they lied to than those in the honest groups, consistent with the findings of previous studies (Li et al., 2021a,b). This result suggests that liars might forget who they lied to when they have told many lies to multiple people. We can also consider this interesting finding a deceive-induced forgetting (DIF) effect in the destination memory. It has been suggested that deception requires cognitive resources (Vrij and Heaven, 1999; Suchotzki et al., 2017; Otgaar and Baker, 2018). The participants in the deception groups were asked to respond deceptively to all interview questions. The items asked about in the interview contained five items on the shopping list and five items not on the shopping list. For the participants in the deception group, too many cognitive resources might be consumed to identify whether the items asked about were on the shopping list, inhibit the truth, construct lies and give deceptive responses. On the other hand, the participants in the honest group might not be needed to consume many cognitive resources but just respond honestly. Therefore, the participants in the deception groups perhaps had fewer cognitive resources to remember the person they lied to than those in the honest groups, which produced a DIF effect in the destination memory test.

We also observed that the participants in the deception group under the high-involvement condition created more non-believed memories than those in the honest group, both in the source memory and destination memory tests. These findings are consistent with those of previous studies (Otgaar et al., 2014b; Polage, 2017; Battista et al., 2020a; Li et al., 2021a,b) and suggest that lying impairs a liar's memory and belief and leads to more uncertain memories.

The findings from this study have some theoretical and practical implications. First, in the deception and memory framework, Otgaar and Baker (2018) proposed that lying requires cognitive resources and that the cognitive resource requirements vary with the type of lie. The more cognitive resources a lying strategy requires, the more impairments it will cause. A number of studies have provided positive evidence for this proposition (Mangiulli et al., 2018, 2019, 2020; Otgaar et al., 2018b; Battista et al., 2021b). However, the present study suggests that the effects of deception on memory are also modulated when liars with different degrees of involvement adopt the same lying strategy. Another study had also found that the effects of deception on memory differed when the participants employed the same deceptive strategy (false denial) (Battista et al., 2021a). Therefore, the key factor modulating the effects of deception on memory may not be the types of deception strategies. The consumption of cognitive resources and the type of deception strategy may not correspond exactly. The situation in real life is very complicated, and we cannot directly predict the disruptions that their memories are suffering from the strategies used by liars. Moreover, the cognitive resource is a general concept, and specific basic cognitive mechanisms need to be considered in this field to clarify what plays the key role in the effects of deception on memory. In the present study, we preferred the inhibition theory rather than the theory of cognitive resource consumption for the DIF effect observed in the source memory test. It has been argued that inhibition is one of the basic cognitive mechanisms (Bond, 2012). The truth may be activated automatically, and liars who give false denial responses may need to invert the truth. To make a successful truth inversion, liars may need to inhibit the truth first. The participants in the high-involvement condition got much more information than those in the low-involvement condition during shopping, and then the former might be needed to make more effort to inhibit information in the interview than the latter ones. On the other hand, the participants in the honest groups did not need to inhibit anything in the interview. Thus, the effects of deception on memory differed in and modulated by the degrees of involvement. Comparing the studies that obtained a DIF effect in the source memory test with those that did not, we suggest that some variables, such as tasks and individual differences, may also play a role in producing a DIF effect. Additional studies on this issue need to be undertaken before the association between false denial and the DIF effect is more clearly understood. Second, consistent with previous studies (Li et al., 2021a,b), the present study also found that liars forgot who they lied to, and the liars who were highly involved in the events also forgot what they lied about and had more non-believed memories for what they lie about and to whom. Therefore, it is not safe for their lies when people involve in an event and tell lies to different people about the event. Moreover, when liars hear a true account of an event from someone else and lie to other people, they might remember the lies they tell but also forget who they lied to. Liars may be uncovered because it is not easy for them to remember what they lied about and who they lied to. Thus, it is dangerous when people tell many lies and lie to multiple people in a short period of time.

A limitation of this study is needed to be acknowledged. It has been suggested that females and males differ in their lying behaviors in daily life (DePaulo et al., 1996) and in their deceptive neural responses (Marchewka et al., 2012; Gao et al., 2018). However, most participants were female, with <20% of the participants being male in the present study. Therefore, the small number of males among the participants requires caution when generalizing these findings. Future work needs to be done to establish whether there are sex differences in the effects of deception on memory.

In summary, the present study was designed to determine whether involvement modulates the effects of deception on memory using a daily-life paradigm. The most obvious finding to emerge from this study is that liars in the high-involvement condition lost more memories and had more non-believed memories in the source memory test and the destination memory test, and those in the low-involvement condition forgot who they lied to. Moreover, a DIF effect was found in the source memory test in the high-involvement condition but not in the low-involvement condition. Thus, the results indicate that involvement modulates the effects of deception on memory in daily life.

## Data Availability Statement

The raw data supporting the conclusions of this article will be made available by the authors, without undue reservation.

## Ethics Statement

The studies involving human participants were reviewed and approved by Faculty of Psychology at Tianjin Normal University. The patients/participants provided their written informed consent to participate in this study.

## Author Contributions

YL: introduction, methodology, and first draft preparation. ZL: introduction, methodology, and final preparation. All authors contributed to the article and approved the submitted version.

## Conflict of Interest

The authors declare that the research was conducted in the absence of any commercial or financial relationships that could be construed as a potential conflict of interest.

## Publisher's Note

All claims expressed in this article are solely those of the authors and do not necessarily represent those of their affiliated organizations, or those of the publisher, the editors and the reviewers. Any product that may be evaluated in this article, or claim that may be made by its manufacturer, is not guaranteed or endorsed by the publisher.
